# Relapsed Immune Thrombocytopenic Purpura Following COVID-19 Vaccination in a Patient With Autoimmune Cytopenias

**DOI:** 10.14740/jmc5305

**Published:** 2026-04-29

**Authors:** Ella Lahart, Linda Akbarshahi, Mariya Barbarovich, Eric Evans

**Affiliations:** a GME Research Department, Northeast Georgia Medical Center, Gainesville, GA, USA; b Family Medicine, Northeast Georgia Medical Center, Gainesville, GA, USA

**Keywords:** Immune thrombocytopenia, Autoimmunity, Immunoglobulins, Intravenous, Glucocorticoids

## Abstract

Immune thrombocytopenic purpura (ITP) is a rare autoimmune condition characterized by isolated thrombocytopenia in the absence of an identifiable secondary cause. While most cases respond to first-line therapy, relapsed or refractory forms, particularly in patients with coexisting autoimmune disorders, pose significant diagnostic and treatment challenges. Recent reports have identified rare instances of autoimmune cytopenias following severe acute respiratory syndrome coronavirus 2 (SARS-CoV-2) mRNA vaccination, raising concern for potential immune reactivation in predisposed individuals with prior immune dysregulation. We present the case of a 68-year-old male with a history of ITP and autoimmune hemolytic anemia who developed a severe relapse of thrombocytopenia, epistaxis, and suspected gastrointestinal hemorrhage shortly after coronavirus disease 2019 (COVID-19) vaccination. Despite initial treatment with corticosteroids, intravenous immunoglobulin, and platelet transfusions, the patient experienced a second relapse requiring escalation of care with additional immunosuppressive therapy and close hematologic follow-up. Ultimately, his condition improved with a multimodal approach and coordinated outpatient management. This case is clinically significant due to its temporal association with COVID-19 vaccination and the relapsing nature of ITP in the setting of systemic autoimmunity. It underscores the importance of early recognition, timely intervention, and vigilant monitoring in preventing life-threatening complications. The report adds to a growing body of literature highlighting the need for individualized care strategies and post-vaccine surveillance in patients at heightened risk of autoimmune reactivation.

## Introduction

Immune thrombocytopenic purpura (ITP) is an acquired autoimmune disorder characterized by isolated thrombocytopenia (platelet count < 100 × 10^9^/L) in the absence of a clear secondary cause [1]. The pathophysiology involves the formation of antiplatelet autoantibodies, most commonly against glycoprotein IIb/IIIa and Ib/IX, leading to premature platelet clearance by the reticuloendothelial system, as well as impaired megakaryocyte function and platelet production in the bone marrow [1]. In adults, ITP affects approximately 3.3 per 100,000 individuals annually in the United States, with incidence increasing with age [2]. Older adults, especially men, tend to present more frequently with relapsed or secondary forms of the disease and may have a more refractory clinical course [2].

Clinically, ITP may range from incidental thrombocytopenia detected during routine bloodwork to severe mucocutaneous, gastrointestinal, or even intracranial hemorrhage [3]. Common manifestations include petechiae, purpura, gingival bleeding, epistaxis, and, in some cases, menorrhagia or hematuria [3]. The degree of thrombocytopenia does not always correlate linearly with bleeding risk, particularly in chronic or relapsed disease [3]. Management is typically initiated in symptomatic patients or when platelet counts fall below 30 × 10^9^/L [3]. First-line treatment includes corticosteroids (e.g., prednisone or dexamethasone), intravenous immunoglobulin (IVIG), or anti-D immunoglobulin in select populations [3, 4]. While many patients achieve remission with initial therapy, up to 30% experience relapse or progress to chronic ITP, requiring long-term immunosuppression, thrombopoietin receptor agonists (thrombopoietin receptor agonists (TPO-Ras)), rituximab, or splenectomy [4].

The complexity of ITP increases when it occurs in the context of systemic autoimmunity or as part of Evans syndrome, a rare hematologic disorder defined by the simultaneous or sequential occurrence of ITP and autoimmune hemolytic anemia (AIHA) [5]. Evans syndrome carries a poorer prognosis and greater risk for relapse, often necessitating prolonged or second-line immunosuppressive regimens [5]. Patients with Evans syndrome also have a higher likelihood of underlying immune dysregulation, including common variable immunodeficiency or systemic lupus erythematosus, which further complicates treatment planning [5].

Recent literature has drawn attention to cases of *de novo* or relapsing autoimmune cytopenias following vaccination, particularly after severe acute respiratory syndrome coronavirus 2 (SARS-CoV-2) mRNA vaccines [6]. Although the absolute incidence is low, growing evidence suggests that vaccinations may act as immune triggers in predisposed individuals, especially those with a history of autoimmune disease [6]. These rare events are thought to reflect a bystander immune activation or molecular mimicry, potentially leading to the reactivation of underlying immune cytopenias [7]. While causality remains difficult to establish definitively, such reports emphasize the importance of close post-vaccine monitoring in patients with known autoimmune hematologic disorders [7].

Here, we present the case of a male in his late 60s with a prior diagnosis of ITP and AIHA who presented with a severe relapse of thrombocytopenia, epistaxis, and suspected gastrointestinal hemorrhage in late 2024. His disease course was complicated by steroid dependence and a second episode of refractory thrombocytopenia following initial hospital discharge. This case illustrates the diagnostic and therapeutic challenges in managing relapsed ITP in patients with complex autoimmune comorbidities and underscores the importance of timely immunosuppressive therapy, multidisciplinary coordination, and vigilant follow-up to mitigate life-threatening complications.

## Case Report

A 68-year-old male presented to the emergency department in late 2024 with a persistent epistaxis, a widespread petechial rash affecting the oral mucosa, torso, and upper extremities, and suspected melena. He denied experiencing associated symptoms, such as fatigue, fever, or other systemic symptoms. The patient had a significant history of ITP and AIHA diagnosed in 2021, which had previously responded well to corticosteroid therapy. The patient had a prior episode of autoimmune cytopenia in 2021 following coronavirus disease 2019 (COVID-19), presenting with similar bleeding symptoms and responding to corticosteroid therapy. While a direct causal relationship between that episode and the current presentation cannot be definitively established, this history suggests a possible predisposition to immune reactivation. There had been no recent infections, medication changes, or known toxin exposures before this presentation. Before arriving at the emergency department, he was evaluated at an urgent care clinic for his recurrent epistaxis. He underwent nasal cauterization by an ear, nose, and throat (ENT) specialist, but the bleeding continued. He also developed new onsets of rash and darker stools during that time. Based on these evolving symptoms, ENT recommended immediate transfer to the emergency department for further evaluation.

On physical examination, the patient exhibited diffuse petechiae on the upper and lower extremities, as well as in the oral mucosa. There was mild mucosal bleeding from the nares and mouth. No rectal examination was conducted due to location constraints, but gastrointestinal bleeding was suspected based on patient history and reported stool changes. Initial bloodwork revealed a platelet count of 0 × 10^9^/L, confirming severe thrombocytopenia. Additional evaluation was performed to exclude alternative causes of severe thrombocytopenia. There was no clinical or laboratory evidence of active hemolysis, and the patient’s hemoglobin remained stable throughout admission. No signs of infection were identified, and leukocytosis observed during subsequent admissions was attributed to corticosteroid therapy. Coagulation studies and metabolic panel were within normal limits, and there was no clinical concern for disseminated intravascular coagulation or thrombotic microangiopathy. These findings, in conjunction with the patient’s history of ITP, supported the diagnosis of relapsed immune thrombocytopenia. Given the critically low platelet count and bleeding symptoms, hematology and oncology specialists were urgently consulted. A presumptive diagnosis of relapsed ITP was made, and treatment was initiated with IVIG at a dose of 1 g/kg (total dose: 70 g), intravenous methylprednisolone 40 mg daily, and two units of platelet transfusions.

During the first 48 h of admission, the patient’s platelet count remained at 0 × 10^9^/L, despite transfusions, IVIG and corticosteroid therapy, indicating a poor initial response to treatment. He continued to experience epistaxis and petechiae, with no other signs of bleeding and no evidence of major internal hemorrhage. An additional two units of platelets were transfused, with minimal immediate effect. By hospital day 3, following continued immunosuppressive therapy, his platelet count began to rise, reaching 30 × 10^9^/L, and mucosal bleeding had subsided. The patient remained stable and was discharged 4 days after admission, with a prescription for oral prednisone 60 mg daily, to be tapered over 6 weeks. He was advised to avoid high-risk activities, trauma, and a hematology follow-up 2–3 weeks later.

The patient was re-presented to the emergency department a week after initial discharge with recurrent thrombocytopenia, epistaxis, petechial rash, severe headache, worsening fatigue, and shortness of breath over the past 3 days. He also reported a new onset of bradycardia, with documented heart rates below 40 beats per minute. A computed tomography (CT) scan of the brain was performed, which was negative for intracranial hemorrhage. Repeat laboratory testing revealed a platelet count of 3 × 10^9^/L, suggesting a relapse or incomplete response to previous treatment. Physical examination revealed petechiae on the upper extremities, torso, and oral mucosa. Mild swelling of the right arm was noted, though no signs of active bleeding were evident. The patient was managed with one unit of platelet transfusion, IVIG (Privigen) was administered at 1 g/kg for two doses, alone with an initial intravenous dexamethasone 10 mg, followed by tapering doses. The response to platelet transfusion alone was limited; however, following combined therapy with IVIG and corticosteroids, the patient demonstrated a favorable response, with platelet counts increasing to 62 × 10^9^/L over the first 48 h of admission. He was discharged with a resolution of his headache and stabilization of mucosal symptoms. He was advised to report any recurrence of bleeding, fatigue, or neurologic symptoms immediately. Pain management was addressed with acetaminophen or prochlorperazine as needed for residual headaches. At his follow-up visit with hematology, laboratory tests showed continued improvement in platelet counts, and he remained clinically stable. Plans for continued tapering of prednisone and hematology oversight were maintained. As of the 2-week post-discharge period, the patient had no relapse of symptoms or need for readmission.

## Discussion

This case is clinically significant due to the relapsed ITP that occurred in the context of a recent COVID-19 mRNA vaccination, coupled with the challenge of managing a patient with an autoimmune background and prior history of Evans syndrome. The recurrence of severe thrombocytopenia, epistaxis, petechial rash, and mucosal bleeding despite initial standard therapy highlights the complexity of treatment and the need for a multimodal, carefully coordinated approach to care.

While ITP is often considered a benign, self-limited disease in many adults, a subset of patients develops chronic or relapsed forms that prove refractory to initial therapy [1]. This patient, with a background of AIHA and prior ITP, fits into a higher-risk group known to experience frequent relapses and less predictable treatment responses [8]. In such patients, the pathogenesis involves both increased platelet destruction due to autoantibodies and impaired megakaryocyte function in the bone marrow, resulting in reduced platelet production [1, 8]. Notably, this patient demonstrated a relapsing and refractory disease course, requiring multiple hospital admissions within a short time frame despite standard first-line therapies. His limited and transient response to platelet transfusions further supports the immune-mediated destruction characteristic of ITP. The need for escalation to second-line therapy, including planned rituximab, highlights the importance of recognizing patients at risk for chronic or refractory disease and initiating timely advanced therapies.

Recent reports have raised concern about autoimmune hematologic disorders arising *de novo* or reactivating after SARS-CoV-2 vaccination, particularly with mRNA-based vaccines [6]. Although causality remains uncertain, a temporal association has been observed in several cases, and a growing body of literature suggests that immune stimulation, either through molecular mimicry, bystander activation, or cytokine dysregulation, may play a role [6]. A systematic review by Lee et al (2022) identified over 70 published cases of ITP following COVID-19 vaccination, with the majority occurring within 14 days of vaccine administration and predominantly in individuals with predisposing autoimmune conditions [9]. However, such events remain extremely rare, with an estimated incidence of 0.80 cases per million doses for mRNA vaccines [6]. Thus, while a causal link cannot be definitively established in this case, the clinical pattern, absence of alternative triggers, and recurrence timeline raise reasonable suspicion of a vaccine-associated flare [6]. Several similar cases of immune thrombocytopenia following SARS-CoV-2 vaccination have been reported in the literature. A summary of selected cases is provided in Table 1 [6, 7, 9–11].

**Table 1:** Summary of Selected Reported Cases of Immune Thrombocytopenia Following SARS-CoV-2 Vaccination

Study	Age/sex	Vaccine type	Time of onset	Prior ITP/autoimmunity	Treatment	Outcome
Lee et al, 2021 [7]	72/F	Pfizer	5 days	No	IVIG and steroids	Platelet recovery
Welsh et al 2021, [6]	65/M	Moderna	10 days	No	IVIG and steroids	Improved
Lee et al, 2022 [9]	68/M	Pfizer	7 days	Yes (ITP)	IVIG and steroids	Relapsed, required retreatment
Tarawneh et al, 2021 [10]	22/F	Pfizer	14 days	No	IVIG and steroids	Recovered
Chong et al, 2021 [11]	75/M	Moderna	3 days	Yes (autoimmune disease)	IVIG and steroids	Partial response

Time of onset refers to the interval between vaccination and onset of thrombocytopenia. Outcomes reflect platelet recovery or clinical response as reported in the original studies. SARS-CoV-2: severe acute respiratory syndrome coronavirus 2; F: female; M: male; IVIG: intravenous immunoglobulin; ITP: immune thrombocytopenic purpura.

Management of relapsed ITP, particularly in complex autoimmune patients, requires a nuanced and often stepwise approach. Standard first-line treatments include corticosteroids (e.g., prednisone or dexamethasone) and IVIG, both of which were utilized in this patient with partial response [11]. However, failure to sustain platelet recovery is common in relapsed cases, necessitating escalation to second-line therapies such as rituximab, thrombopoietin receptor agonists (e.g., eltrombopag, romiplostim), or immunosuppressants like azathioprine or mycophenolate [2, 11]. In this case, the patient ultimately responded to a multimodal strategy of repeated IVIG, high-dose steroids, and transfusions for symptomatic management. Importantly, platelet transfusions are generally reserved for cases of life-threatening hemorrhage, as the underlying autoimmune mechanism renders them transient and often ineffective without concurrent immunosuppression [12]. The chronicity and relapsing nature of ITP, especially in patients with Evans syndrome, also present significant challenges in follow-up care [13]. Close monitoring of platelet counts, bleeding symptoms, and potential drug toxicities is critical, along with tailored tapering schedules to minimize rebound cytopenia [13]. Multidisciplinary care, encompassing hematology, primary care, and occasionally immunology, is crucial for optimizing long-term outcomes [13].

In summary, this case illustrates the complexity of managing relapsed ITP in a patient with a known autoimmune predisposition and recent COVID-19 vaccination. It exemplifies the importance of prompt recognition, the use of a multimodal treatment strategy, and the need for heightened clinical suspicion of autoimmune reactivation in susceptible populations. While the benefits of vaccination overwhelmingly outweigh the risks, this case underscores the necessity of personalized care in individuals with prior autoimmune cytopenias.

### Conclusions

This case is notable for illustrating a suspected vaccine-associated relapse of ITP in a patient with a known history of autoimmune cytopenias. The temporal relationship between COVID-19 vaccination and the recurrence of severe thrombocytopenia, along with the patient’s positive response to a timely, multimodal treatment approach, makes this report clinically relevant. It emphasizes the need for heightened clinical awareness of autoimmune disease flares following immunization, particularly in patients with preexisting immune dysregulation. Key lessons from this case include the importance of early recognition and prompt initiation of treatment to prevent serious complications such as mucocutaneous or internal bleeding. It also highlights the necessity for continued monitoring after hospital discharge, as patients with relapsed ITP remain at risk for recurrence despite initial improvement. Clinicians should maintain a high index of suspicion for vaccine-related immune events in similar cases and advocate for individualized follow-up strategies.

### Learning points

Relapsed ITP in patients with underlying autoimmune conditions such as Evans syndrome can present with severe and refractory thrombocytopenia requiring a multimodal treatment approach. SARS-CoV-2 vaccination may act as a potential trigger for autoimmune cytopenia relapse in predisposed individuals, although a definitive causal relationship remains uncertain. This case highlights the importance of early recognition, prompt initiation of immunosuppressive therapy, and close post-discharge monitoring to prevent life-threatening bleeding complications. It also underscores the need for individualized care strategies in patients with recurrent or complex autoimmune cytopenia.

## Data Availability

All data relevant to this case report are included within the article. Additional information may be made available upon reasonable request.

## Abbreviations

AIHA: autoimmune hemolytic anemia
CT: computed tomography
ENT: ear, nose, and throat
ITP: immune thrombocytopenic purpura
IVIG: intravenous immunoglobulin
SARS-CoV-2: severe acute respiratory syndrome coronavirus 2
TPO-Ras: thrombopoietin receptor agonists
